# Lost in the City: Revisiting Milgram's Experiment in the Age of Social Networks

**DOI:** 10.1371/journal.pone.0111973

**Published:** 2014-11-10

**Authors:** János Szüle, Dániel Kondor, László Dobos, István Csabai, Gábor Vattay

**Affiliations:** Dept. of Physics of Complex Systems, Eötvös Loránd University, Budapest, Hungary; Universitat Pompeu Fabra, Spain

## Abstract

As more and more users access social network services from smart devices with GPS receivers, the available amount of geo-tagged information makes repeating classical experiments possible on global scales and with unprecedented precision. Inspired by the original experiments of Milgram, we simulated message routing within a representative sub-graph of the network of Twitter users with about 6 million geo-located nodes and 122 million edges. We picked pairs of users from two distant metropolitan areas and tried to find a route between them using local geographic information only; our method was to forward messages to a friend living closest to the target. We found that the examined network is navigable on large scales, but navigability breaks down at the city scale and the network becomes unnavigable on intra-city distances. This means that messages usually arrived to the close proximity of the target in only 3–6 steps, but only in about 20% of the cases was it possible to find a route all the way to the recipient, in spite of the network being connected. This phenomenon is supported by the distribution of link lengths; on larger scales the distribution behaves approximately as 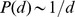, which was found earlier by Kleinberg to allow efficient navigation, while on smaller scales, a fractal structure becomes apparent. The intra-city correlation dimension of the network was found to be 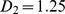, less than the dimension 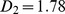 of the distribution of the population.

## Introduction

Small-world phenomenon [1] has been a focus of research of social sciences for decades. While many different types of random graphs exist with the property of having short path lengths between pairs of nodes [2], [3], finding these nodes, in general, can prove hard and usually requires global information about the network. Geographically embedded networks, on the other hand, usually make it possible to find routes between pairs of nodes based on local information only, a property called navigability [4]. In the original experiments of Milgram [1], [5], randomly chosen individuals living in the mid-western USA were asked to deliver a letter to a given address in Boston but they were only allowed to forward it directly to personal acquaintances. Although the results were widely popularized as the phenomenon of *six degrees of separation*, in reality, the mean length of shortest paths between persons in social networks is even less [6]. By examining the strategy of the individuals taking part in the experiment, Travers & Milgram found that people were most likely to forward the letter to an acquaintance thought to be living the closest to the target address.

With the increasing availability of social network data, the results of Travers & Milgram gained revived attention. Dodds & Watts investigated models of routing on social networks [7] and carried out experiments similar to that of Travers & Milgram using email communication instead of letters [8]. In his seminal paper, Kleinberg showed theoretically [4], [9] for an important family of random networks embedded into a metric space that the simple *greedy routing* algorithm is efficient to find paths between nodes when the link lengths of the network are distributed according to the power-law 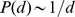. Previous simulations carried out on real social network data confirmed Kleinberg's results [10] and his model was generalized to more complex models of networks [11], [12]. Other studies found very similar link length distributions in various kinds of real-world networks [10], [13]–[17]. Further studies found that networks with this kind of link length distribution are special in other aspects as well [18]–[20].

Online social networks have become a major phenomenon during the last decade, and certain service providers, like Twitter, make their data partially or completely available to the public which offers new data mining opportunities for social and network scientists. The available data have made studying many important questions possible [21]–[27] which could not be addressed before.

## Analysis

In this paper we present the results from our simulation of greedy routing on the geographically embedded social network of Twitter users. Our data set was compiled from a database of over three billion short messages (tweets) collected from the freely available “sprinkler” stream of Twitter over a course of sixteen months [28]. We identified over sixteen million individuals broadcasting GPS coordinates regularly along with their tweets, and selected the most active six million users for this study. In the Twitter social network, users can establish one-way connections by *following* the tweets of others. To find the most active users, tweeters were sorted by the number of followers multiplied by the number of their own tweets. We discovered the follower graph of the most active users and considered two individuals being *friends* only if they follow each other mutually. This resulted in an undirected graph with 5.8 million nodes and 122 million edges, with 99.3% of the nodes being in a connected sub-graph. Since we are interested in finding routes between the users, we first examined the shortest paths in the network using breadth-first search. From a random sample of 80,000 user pairs, we estimated the average separation to be 4.95 hops. To be able to simulate greedy routing, we required a single representative location for each user. Since a single user can have as many as a few hundred different GPS coordinates, we applied the friend-of-friend clustering technique to identify the largest clusters of coordinates which we consider representative locations of the user. To find the average coordinate of each cluster, we iteratively pruned the data points until all GPS coordinates of a cluster fell into a 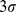 radius of the average. Data collection and preprocessing are described in more detail in a former paper by our research group [28].

To run simulated message routing experiments on the network of Twitter friends, we implemented Kleinberg's greedy routing algorithm [4], [9] with minor modifications. To find a route between two randomly chosen individuals with known geographic coordinates, we hop from a node to its neighboring network node closest to the target in terms of spatial separation (great circle distance). In Kleinberg's original model, the routing was always successful (although sometimes slow) thanks to the underlying grid connecting the individuals. Since our graph does not have such regular structure, in our case, infinite loops and dead ends would be possible. To avoid these, we keep track of already visited nodes and never choose a node which has been visited before. If the current node is surrounded by visited nodes only, we abort the routing and consider it unsuccessful. Also, we limit the maximum length of a route by considering it unsuccessful if the target could not be reached in 100 steps. The number 100 is well within the long tail of the hop distribution, as we will show later. We repeated the simulation two ways. In the first case, we chose the source and target individuals randomly from the entire world and repeated the process 4 million times. In the other cases, we picked 2000–2000 individuals randomly from selected urban areas and simulated the routing in all possible combinations, again totaling in 4 million routes. This approach allowed us to compare the effectiveness of greedy routing between societies with different Twitter usage habits.

Kleinberg in his original theoretical work defined the navigability of a geographically embedded network as a network on which greedy routing is efficient. The simplest greedy routing algorithm is especially appealing, from both the theoretical and the empirical point of view, as it relies on local connectivity and geographic information about the closest neighbors only. Obviously, more elaborate, heuristic algorithms than greedy routing exist to navigate networks more efficiently, but these algorithms often depend on information other than geographic coordinates of the immediate neighborhood [29]. While these algorithms are certainly useful to investigate the connections between other network properties and navigability, however, these are beyond the scope of the current study.

**Figure 1:**
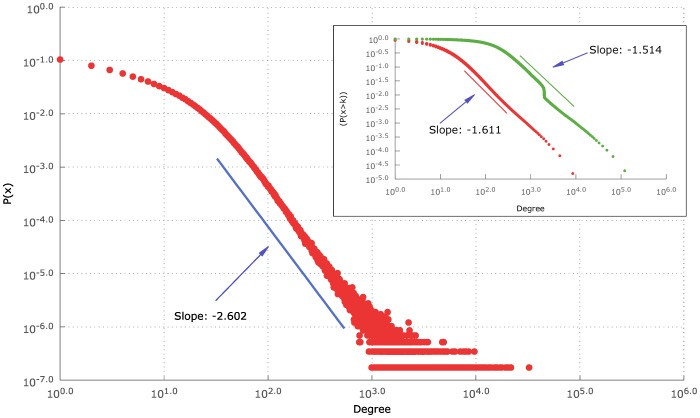
Degree distribution of the Twitter friendship graph. Main plot: Degree distribution within the friendship network. In the inset: cumulative degree distribution in the friendship network (red) and out-degree distribution of the users not restricted to the 5.8 million sample (green). The jump in the green curve is due to Twitter policy of limiting follows up to 2000 users in case of a disproportionate in and out degree ratio [34].

Boguna et al. [30] showed on simulated data that the success rate of greedy routing in a navigable network depends mainly on the degree distribution 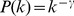 and the clustering coefficient 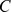. According to them, when the exponent 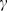 of the degree is approximately 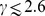 and 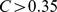 then the probability of finding a path between two points using greedy routing is maximal. We plot the distribution of degrees for the geo-tagged Twitter users on Fig. 1. The distribution is scale-free between 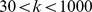 and the exponent turned out to be 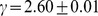. The clustering coefficient is 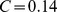, which is clearly outside the region of navigability, as determined by Boguna et al.

It needs to be mentioned that the clustering coefficient of the graph varies significantly from region to region, for example, its value for Turkey and Venezuela is 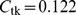 and 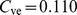, respectively, while it is 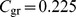 for Greece and 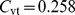 for the US state of Vermont. This might not be entirely due to variations in the distribution of the population but also because of the fact that Twitter usage patterns (especially of users who disclose their GPS coordinates) vary by geographic region.

**Figure 2:**
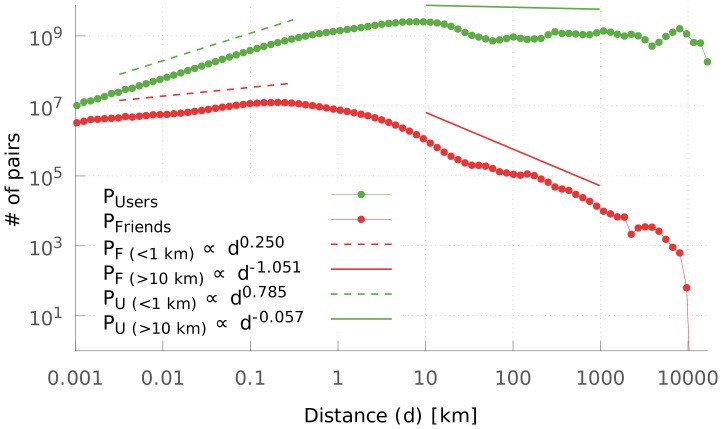
Distance distribution among Twitter users. Probability density distribution of distances between Twitter *friends* (red) and between any two users in the data set (green). The 1 meter–1 kilometer range can be characterized with fractal dimensions 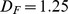 and 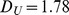 respectively. On the 10 km–1000 km range the friend distance distribution shows an approximate 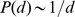 scaling. The figure is based on 5.8 million geo-tagged users worldwide, 122 million friendships and 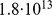 user distances.

To demonstrate that the distribution of link lengths plays a key role in the navigability of spatially embedded networks, we plot the histogram of distances on Fig. 2 between all possible pairs of users, and between friends only. For long-range connections (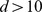 km) the distribution of link lengths of the friends network is well approximated by the power-law 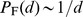. The exponent of 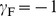 is a necessary condition of navigability [4], [10], [13]–[17]. This means that while most links are short range, there are enough long range connections to enable rapid spreading of information to geographically distant regions. In case of intra-city link lengths (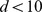 km), on the other hand, the situation is entirely different. The exponent of the power-law fitting the link length distribution of the network of friends for short-range links is 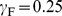. This means that the network of friends shows fractal nature on small scales with a correlation dimension of 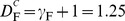 [31]. By comparing 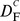 to 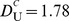, the correlation dimension estimated for the set of coordinates of all users, we can see that the spatial distribution of friends is significantly less steep than the distribution of the whole population [32], [33].

**Figure 3:**
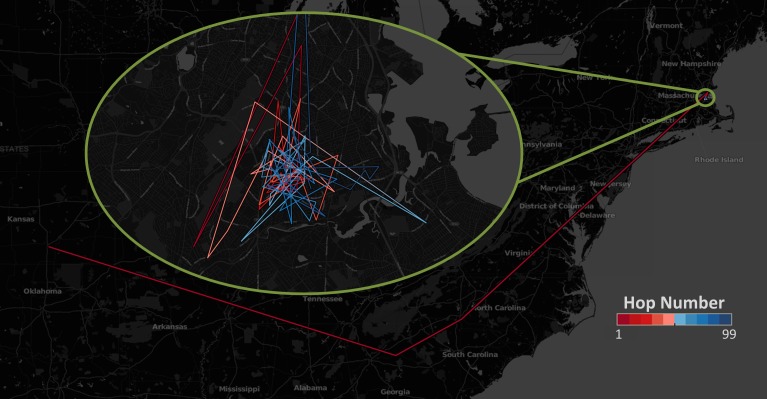
Routing process between two individual twitter users. The initial user has been selected from Wichita, and the target user has been selected from Boston.

In agreement with the behavior of the link length distribution, the navigability of the Twitter social network breaks down below 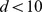 km, the approximate size of a city. To illustrate this, in Fig. 3 we plot a typical journey of a message from a person in Wichita, KS to another person in Boston, MA. One can see that greedy routing can very easily navigate to the geographic neighborhood of the target but then it suddenly gets lost and spends 90% of the time wandering around the neighborhood before it finally reaches its target. This is in accordance with the observation of Travers & Milgram, who also noted that their letters generally reached the neighborhood of their target quickly but, then they would roam around the neighborhood randomly until they found the target's inner circle of friends. This is especially interesting, as in the original Milgram experiment individuals could make decisions on whom to forward the letters based on information other than geographic proximity to the target. These other factors (e.g. occupational proximity) are usually thought to have played a significant role in the decision of the individuals and greatly helped routing on intra-city scales. Testing these factors in our simulation was not possible.

**Figure 4:**
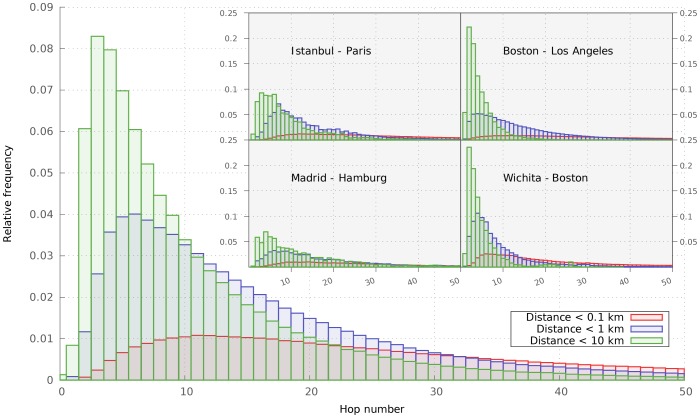
Number of steps needed to reach the proximity of target users. Worldwide distribution of number of steps needed to reach a 0.1,1 and 10 km distance from a designated person. In the inset we show the same statistics for four selected city pairs in the US and Europe.

To examine the effectiveness of greedy routing, we plot the histogram of the number of hops required to approach the target to a given distance in Fig. 4, for random pairs of individuals selected from the entire world. One can clearly see that reaching a target with less geographic accuracy requires significantly less hops on average, and the distribution of the required hops is much less heavy-tailed. The distribution of hops necessary to reach a target within the entire world has a median of 7 and a mode of 4. It is important to mention, however, that on the global scale, only about 20% of simulations reached their targets within 100 steps. A similar success rate was achieved by the original small-world experiment of Milgram, but he attributed the failure of receiving the letters to the lack of motivation of participants. In the light of our results, this might have well been due to the unnavigability of the social network on small scales.

**Figure 5:**
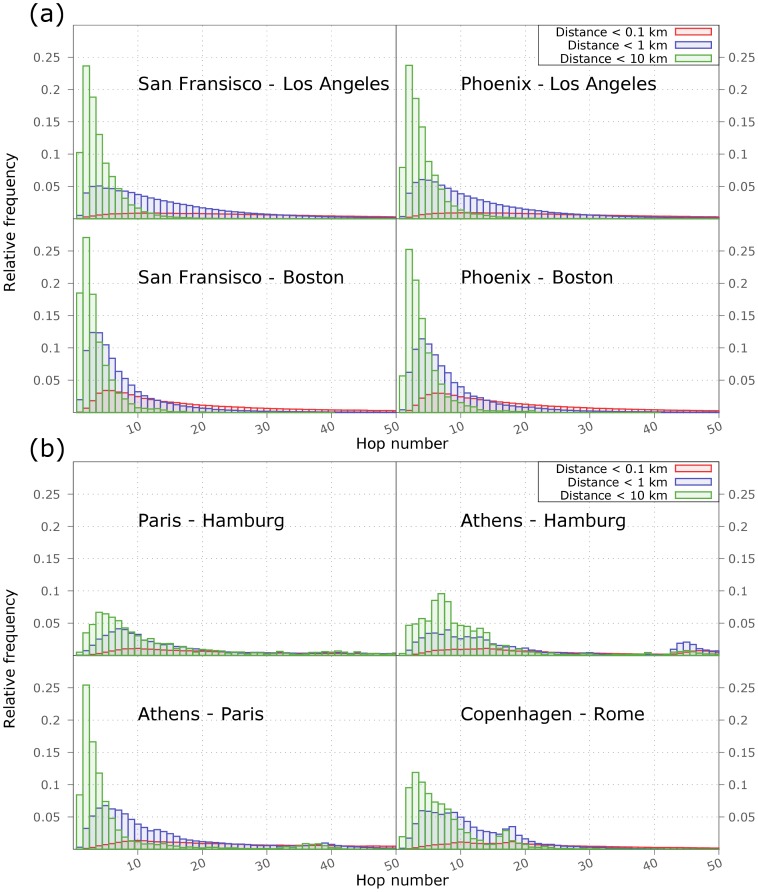
Number of steps needed to reach the proximity of target users. Here we show the difference in the distribution of hop numbers between city pairs selected from USA (*a*) and Europe (*b*). The same statistics has been used like in Fig. 4.

Because different city pairs show different behavior, we plot the histograms of the number of hops necessary to navigate between individuals picked from various urban areas in the USA and in Europe in Fig. 5. By looking at the hop distributions, we can draw a rather interesting conclusion. One is not particularly surprised that, in case of US cities, the effectiveness of routing depends mostly on the target city. This is understandable if we assume that a message finds its way out from any locality in one or two steps. In case of European cities, however, the distributions are dependent on both the origin and the target. While it is very hard to draw universal conclusions from Twitter data due to the different penetration of the platform in different societies, we attribute the observed behavior of routing effectiveness to lingual and cultural barriers among European cities.

## Conclusions

In summary, we have shown that several findings of the original Travers–Milgram experiments [5] can be reproduced by simulated routing on the geo-tagged Twitter network. We found that the network is navigable on inter-city scales using the greedy routing algorithm, and that the close proximity of any target can be reached in only a few steps. This is in accordance with the theoretical results of Kleinberg [4], [9], since the link length distribution on inter-city scale was found to follow the power-law 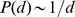 which, in Kleinberg's model of spatially embedded networks, is a necessary condition for efficient navigation. On intra-city scales, on the other hand, navigability breaks down due to the change in the exponent of the link length distribution. The different exponent suggests that the geographic distribution of Twitter users on these distance scales shows some kind of fractal behavior. This is not very surprising since one would expect that geographical distance forms an important role in the establishment of acquaintances on large scales while the effect of distance is much smaller on intra-city scales. As a results, greedy routing works efficiently on the large distance scale where the geographical embedding plays an important role in the network structure, and not on smaller scales where other properties (e.g. occupation or social status) determine the probability of the establishment of social bonds.
